# Handbook of Hospital Practice and Physical Diagnosis

**Published:** 1890-03

**Authors:** 


*Handbook of Hospital Practice and Physical Diagnosis.* By CHRISTOPHER J. NIXON, M.B., LL.D. Univ. Dublin, &c., &c., Senior Physician Mater Misericordiæ Hospital, &c., &c. London: J. & A. CHURCHILL. Dublin: FANNIN & CO. 1889.

Quite recently it has been stated that the clinical teaching in the Dublin hospitals is, or was defective, and that some foundation existed for this indictment seems proved by the fact that the combined Colleges of Physicians and Surgeons of Ireland have now instituted two separate examinations upon the subjects of Hospital Practice of the candidates for their licence. Dr. Nixon has prepared a manual to meet the requirements of students for a guide to physical diagnosis so far as medicine is concerned, a manual of very considerable value. A large part of the work is occupied by the sections pertaining to the examination of the thoracic viscera, the physical signs pertaining to the heart and its associated organs being treated in great detail. Most other topics, however, are also fairly well discussed, such as the pulse, temperature, examination of the urine, fæces, and so forth, each explained with as much detail as a student can be expected to become conversant with. The nervous system and one or two other special subjects are not treated of in this work, remaining to be dealt with separately. The book is adorned with many illustrations borrowed from various sources, and disfigured by many misprints, some of which are comical and some positively misleading. The portions on the microscopical examination of sputum, urinary deposits, and such like, seem to lack authority and precision.

